# Use of extracorporeal membrane oxygenation and associated outcomes in children hospitalized for sepsis in the United States: A large population-based study

**DOI:** 10.1371/journal.pone.0215730

**Published:** 2019-04-26

**Authors:** Katharine Robb, Aditya Badheka, Tong Wang, Sankeerth Rampa, Veerasathpurush Allareddy, Veerajalandhar Allareddy

**Affiliations:** 1 Division of Critical Care, Department of Pediatrics, Stead Family Children’s Hospital, University of Iowa, Iowa City, Iowa, United States of America; 2 Department of Management Sciences, Tippie College of Business, University of Iowa, Iowa City, Iowa, United States of America; 3 Management & Marketing Department, School of Business, Rhode Island College, Providence, Rhode Island, United States of America; 4 Brodie Craniofacial Endowed Chair, Department of Orthodontics, College of Dentistry, University of Illinois at Chicago, Chicago, Illinois, United States of America; 5 Duke University Medical Center, Durham, North Carolina, United States of America; IRCCS Policlinico S.Donato, ITALY

## Abstract

**Objective:**

The American College of Critical Care Medicine recommends that children with persistent fluid, catecholamine, and hormone-resistant septic shock be considered for extracorporeal membrane oxygenation (ECMO) support. Current national estimates of ECMO use in hospitalized children with sepsis are unknown. We sought to examine the use of ECMO in these children and to examine the overall outcomes such as in-hospital mortality, length of stay (LOS), and hospitalization charges (HC).

**Methods:**

A retrospective analysis of the National Inpatient Sample, which approximates a 20% stratified sample of all discharges from United States community hospitals, was performed. All children (≤ 17 years) who were hospitalized for sepsis between 2012 and 2014 were included. The associations between ECMO and outcomes were examined by multivariable linear and logistic regression models.

**Results:**

A total of 62,310 children were included in the study. The mean age was 4.2 years. ECMO was provided to 415 of the children (0.67% of the cohort with sepsis). Comparative outcomes of sepsis in children who received ECMO versus those who did not included in-hospital mortality rate (41% vs 2.8%), mean HC ($749,370 vs $90,568) and mean LOS (28.8 vs 9.1 days). After adjusting for confounding factors, children receiving ECMO had higher odds of mortality (OR 11.15, 95% CI 6.57–18.92, *p* < 0.001), longer LOS (6.6 days longer, *p* = 0.0004), and higher HC ($510,523 higher, *p* < 0.0001).

**Conclusions:**

Use of ECMO in children with sepsis is associated with considerable resource utilization but has 59% survival to discharge. Further studies are needed to examine the post discharge and neurocognitive outcomes in survivors.

## Introduction

Sepsis is a leading cause of morbidity and mortality among children worldwide [1–5]. In the United States, pediatric sepsis results in more than 75,000 hospitalizations and 6,800 deaths each year [6–9]. Children hospitalized with sepsis have mortality rates of 6–14% [6, 8, 10]. In children with septic shock, however, mortality rates increase to 17% [11]. The factor most strongly associated with increased mortality in sepsis in the development of refractory shock [12, 13].

The American College of Critical Care Medicine (ACCM) defines refractory septic shock as shock that persists despite goal-directed use of inotropes, vasopressors, vasodilators, and maintenance of metabolic and hormonal homeostasis [14]. Recent reports have demonstrated that extracorporeal membrane oxygenation (ECMO) can be a life-saving therapy in patients with refractory septic shock, with survival rates as high as 80% in neonates and nearly 50% in children [5, 14–17]. Single-center studies offer further support for the use of ECMO in refractory septic shock [15, 18], and the 2008 update to the Surviving Sepsis Campaign guidelines as well as recent ACCM recommendations encourage consideration of ECMO for pediatric patients with refractory septic shock [14, 19]. Following those recommendations, a retrospective study of 43 US children’s hospitals demonstrated a 6% ECMO utilization rate in children with severe sepsis, and an accompanying reduction in mortality from 18.9% to 12% [16].

The number of centers providing ECMO increased by 55% between 2009 and 2015, with a concurrent 24% increase in the number of pediatric patients receiving the therapy [17]. Although ECMO use appears to have become more widespread in pediatric septic shock over the past several years, the current national rates of ECMO utilization and related outcomes for children with refractory septic shock are largely unknown [16, 20]. We sought to examine the use of ECMO in children with sepsis and its associated outcomes, including in-hospital mortality, length of stay (LOS), and hospital charges.

## Materials and methods

### National Inpatient Sample database and study design

We performed a retrospective analysis of the National Inpatient Sample (NIS) for the years 2012 to 2014. The NIS is the largest all-payer inpatient healthcare database in the United States and is sponsored by the Agency for Healthcare Research and Quality (AHRQ) as part of the Healthcare Cost and Utilization Project (HCUP) [21]. The NIS is a 20% stratified sample of discharges from hospitals in the United States, and is representative of nearly 100% of hospitalizations occurring each year [21].

### Institutional Review Board approval and data user agreement

The present study was granted Institutional Review Board exempt status from the Office of Human Subjects Protection Office of the University of Iowa since de-identified publicly available datasets were used. *The Federal Regulations 45 CFR 46*.*101 (b) states that “research involving the collection or study of existing data*, *documents*, *records*, *pathological specimens*, *or diagnostic specimens*, *if these sources are publicly available or if the information is recorded by the investigator in such a manner that subjects cannot be identified*, *directly or through identifiers linked to the subjects”*. *Based on this regulation such studies are permitted to be classified as “exempt” from IRB full or expedited review*. This study was a retrospective analysis of AHRQ hospital based discharge dataset that is publicly available for purchase.

We completed a data user agreement with HCUP-AHRQ and obtained the NIS data sets. According to the data-user agreement, individual table cell counts of 10 or lower cannot be presented to preserve patient confidentiality. Consequently, these data were not reported in our study and are represented by the designation DS, for discharge information suppressed.

### Selection of patients, outcome variables, and statistical approach

The NIS contains 30 diagnosis fields. The first diagnosis field, primary diagnosis, identifies the reason for hospitalization. The Clinical Classification Software code for sepsis in the primary diagnosis field was used to select the present study cohort [Healthcare Cost Utilization Project. Clinical classifications software (CCS) for ICD-9-CM. Rockville, MD: Agency for Healthcare Research and Quality https://www.hcup-us.ahrq.gov/toolssoftware/ccs/ccs.jsp]. Using this method, all children up to 17 years of age who were hospitalized for sepsis were identified and included in the analysis.

The primary independent variable was use of ECMO. This was identified using ICD-9-CM procedure codes for ECMO in the corresponding fields of the database [International classification of diseases, ninth revision, clinical modification (ICD-9-CM). Hyattsville, MD: National Center for Health Statistics (NCHS); Available from: https://www.cdc.gov/nchs/icd/icd9cm.htm]. Other variables examined included age, sex, race, insurance status, comorbid burden, type of admission, teaching status/setting of hospital, and geographic region. The NIS comorbid severity files were used to estimate the comorbid burden. A total of 29 conditions were identified: AIDS, alcohol abuse, deficiency anemias, rheumatoid arthritis/collagen vascular diseases, chronic blood loss anemia, congestive heart failure, chronic pulmonary disease, coagulopathy, depression, diabetes—uncomplicated, diabetes—with chronic complications, drug abuse, hypertension, liver disease, lymphoma, fluid and electrolyte disorders, metastatic cancer, neurological disorders, obesity, paralysis, peripheral vascular disorders, psychoses, pulmonary circulation disorders, renal failure, solid tumor without metastasis, peptic ulcer disease excluding bleeding, valvular disease, and weight loss.

The outcome variables of interest included in-hospital mortality, LOS, and hospitalization charges. Charges were inflation-adjusted to year 2014 US dollar values using the Bureau of Labor Statistics inflation calculator [Consumer Price Index (CPI) inflation calculator. Washington, DC: Bureau of Labor Statistics; [cited 2017 Jun 16]. Available from: https://www.bls.gov/data/inflation_calculator.htm]. Since LOS and hospitalization charges were highly skewed, log transformed values were used as the outcome variables in the regression models.

The associations between the independent variables and outcomes were examined using multivariable logistic (for in-hospital mortality) and linear (for log-transformed LOS and hospital charges) regression models. Effects of clustering of outcomes within hospitals were adjusted in all regression models. Variances were computed using the Taylor linearization method, assuming a with-replacement design. All statistical tests of association were two-sided, and a *p*-value of < 0.05 was deemed statistically significant. All statistical tests were performed using SAS (Version 9.4) and SAS Callable SUDAAN (Version 11.0.1) software [Research Triangle Institute, Cary, NC].

## Results

From 2012 to 2014, 62,310 children ≤ 17 years of age were hospitalized for sepsis in the United States (Table 1). These 62,310 patients who were admitted for sepsis represent the entire cohort of 100% of hospitalizations that occurred in the USA over the study period. The mean age of the cohort was 4.2 years. Over half (52.3%) of patients were male. Most patients (47.1%) were white, 25.3% Hispanic, and 16.6% black, with other races constituting the remaining 11%. The in-hospital mortality rate was 3.1% (1,930 patients). Nearly half (46.8%) of the patients did not have any comorbid conditions. ECMO support was provided to a total of 415 patients, or 0.67% of the cohort (1 in 145 of those who had sepsis). Of these, 375 (90%) had only one run, while the remaining 40 (10%) received two or more ECMO runs. The majority of patients (77.9%) were treated in urban teaching hospitals.

**Table 1:** Characteristics of children 0–17 years hospitalized due to septicemia.

Characteristics		Number	Percentage
ECMO	Required ECMO	415	0.67
	Did not require ECMO	61895	99.33
Sex	Male	32615	52.35
	Female	29685	47.65
Race	White	26445	47.14
	Black	9310	16.60
	Hispanic	14185	25.29
	Asian/Pacific Islander	2100	3.74
	Native American	705	1.26
	Other Races	3350	5.97
Insurance	Medicare	295	0.47
	Medicaid	35540	57.11
	Private	21765	34.98
	Uninsured	1710	2.75
	Other insurance	2920	4.69
Median Household Income	Quartile 1 (lowest 25% in United States)	21065	34.47
	Quartile 2	16290	26.66
	Quartile 3	13385	21.90
	Quartile 4 (highest 25% in United States)	10365	16.96
Comorbidities*	0	29140	46.77
	1	16490	26.46
	2	8465	13.59
	3	4510	7.24
	4	2325	3.73
	5	1020	1.64
	6	260	0.42
	7	75	0.12
	≥8	25	0.04
Year of Admission	2012	19220	30.85
	2013	20180	32.39
	2014	22910	36.77
Type of Admission	Emergent/urgent	57665	92.76
	Elective	4500	7.24
Type of Hospital	Rural	4080	6.55
	Urban non-teaching	9695	15.56
	Urban teaching	48535	77.89
Region	Northeast	9030	14.49
	Midwest	13310	21.36
	South	24260	38.93
	West	15710	25.21
Disposition	Routine discharge	50885	81.67
	Discharge with home health care	4660	7.48
	Transfer to short-term hospital	3575	5.74
	Transfer to other type of facility	1105	1.77
	Left against medical advice	75	0.12
	Died during hospitalization	1930	3.10
	Not admitted, discharged alive, destination unknown	75	0.12

*Comorbidities: NIS comorbid severity files were used to estimate the comorbid burden. A total of 29 conditions were identified: AIDS, alcohol abuse, deficiency anemias, rheumatoid arthritis/collagen vascular diseases, chronic blood loss anemia, congestive heart failure, chronic pulmonary disease, coagulopathy, depression, diabetes—uncomplicated, diabetes—with chronic complications, drug abuse, hypertension, liver disease, lymphoma, fluid and electrolyte disorders, metastatic cancer, neurological disorders, obesity, paralysis, peripheral vascular disorders, psychoses, pulmonary circulation disorders, renal failure, solid tumor without metastasis, peptic ulcer disease excluding bleeding, valvular disease, and weight loss.

**Fig 1:**
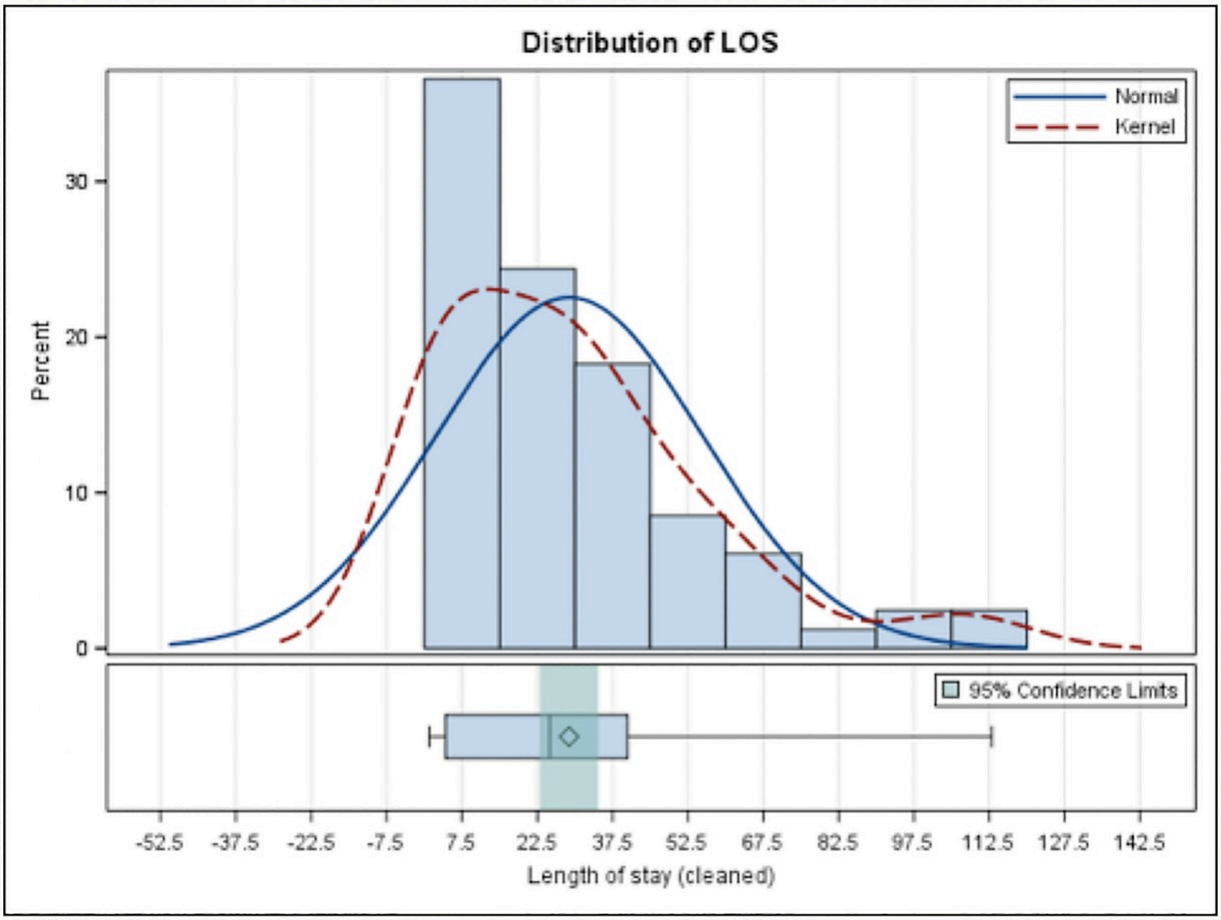
Distribution of LOS in those who had ECMO.

**Fig 2:**
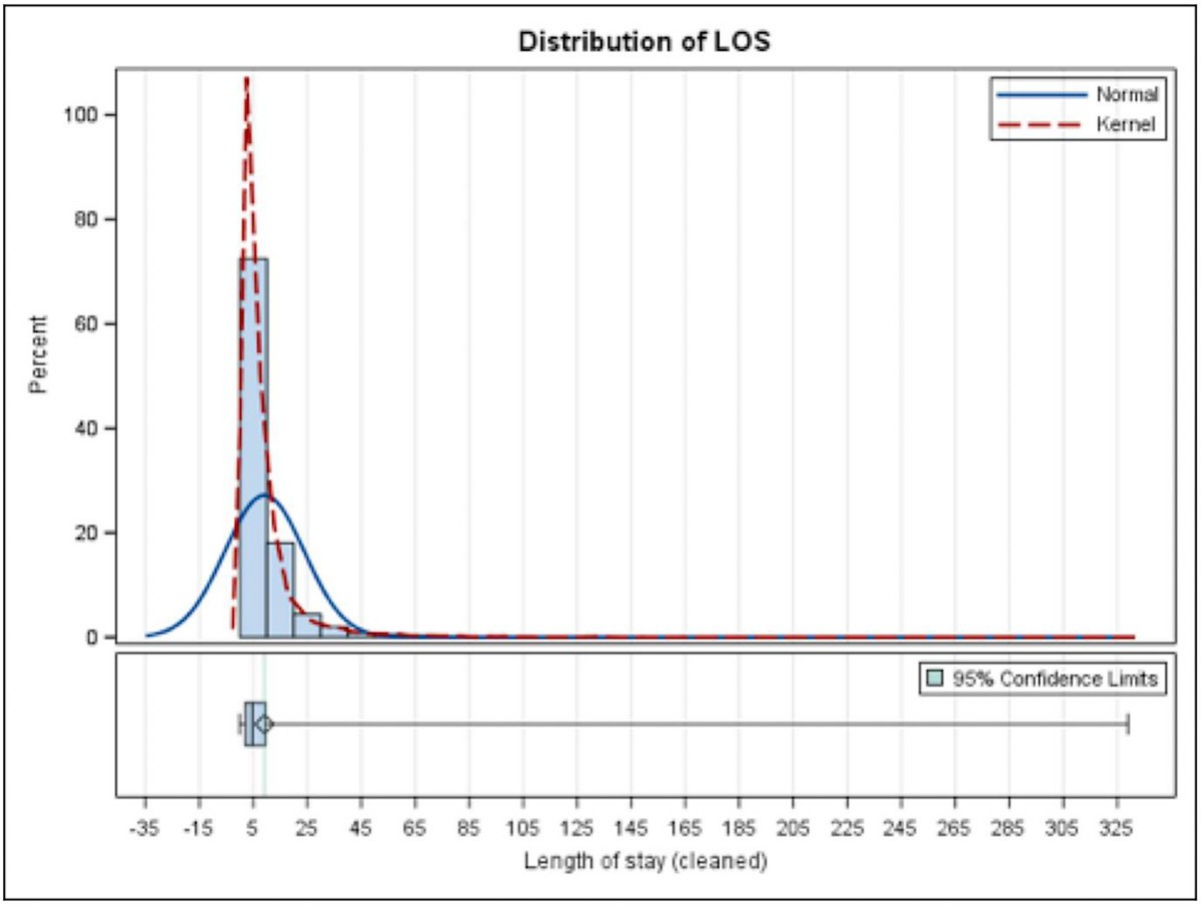
Distribution of LOS in those without ECMO.

**Fig 3:**
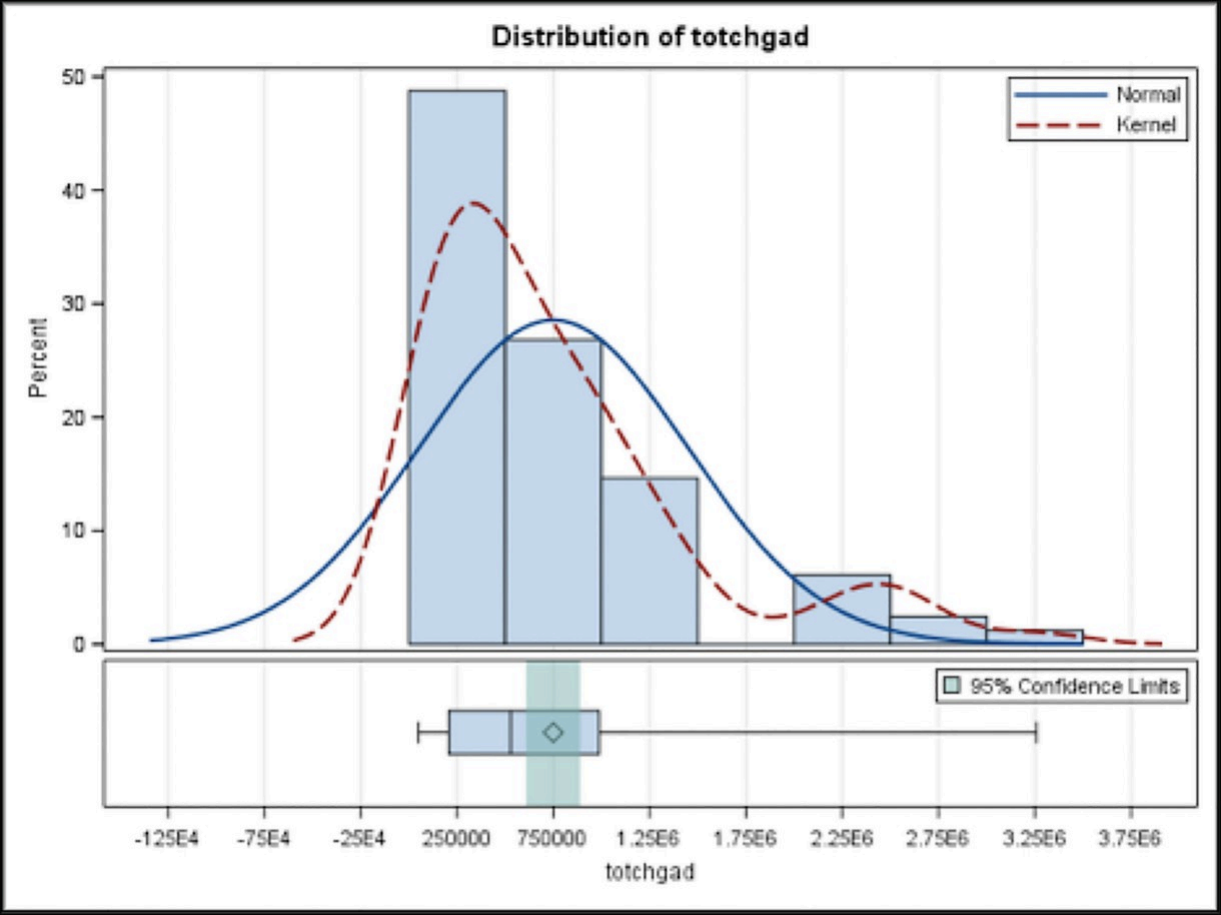
Distribution of Hospital Charges in those who had ECMO, totchgad = Inflation adjusted hospital charges.

**Fig 4:**
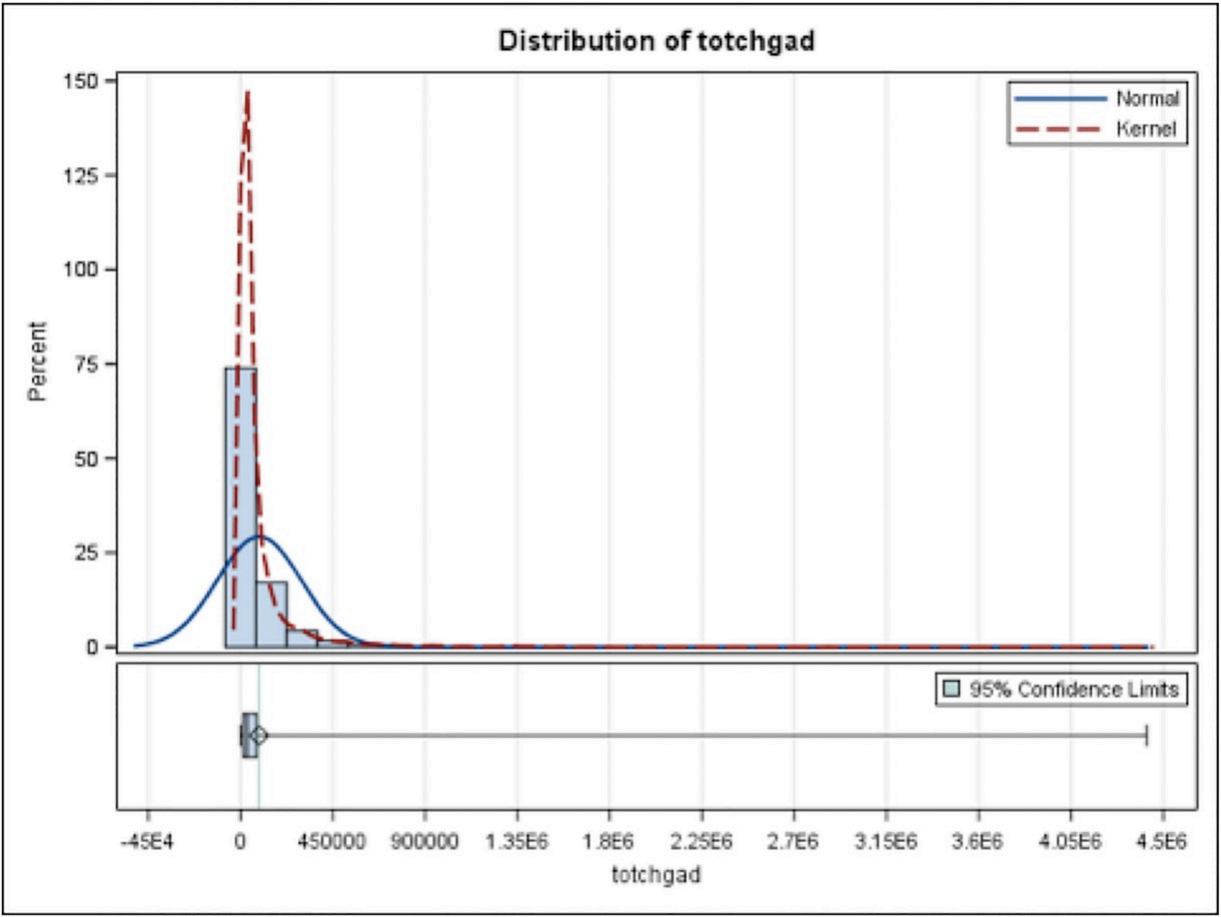
Distribution of Hospital Charges in those who did not have ECMO. totchgad = Inflation adjusted hospital charges.

The hospitalization outcomes are summarized in Table 2. The in-hospital mortality rate was 41% for patients who received ECMO, compared to 2.8% for those who did not. The mean LOS was 28.8 days for those who received ECMO and 9.1 days for those who did not. Distribution of LOS in those who had ECMO and those without ECMO are shown in Fig 1 and Fig 2, respectively. Average hospitalization charges for patients who did and did not require ECMO were $749,370 and $90,568, respectively. Distribution of HC in those who had ECMO and those without ECMO are shown in Figs 3 and 4, respectively.

**Table 2:** Outcomes associated with ECMO use in sepsis.

Outcomes		ECMO	No ECMO	Overall
In-Hospital Mortality	Number	170	1760	1930
	Percentage	41%	2.8%	3.1%
Length of Stay (days)	Mean	28.8	9.1	9.3
	Standard error of mean	2.9	0.19	0.19
	25^th^ percentile	4.2	2.1	2.1
	Median– 50^th^ percentile	25	4.7	4.7
	75^th^ percentile	40.5	9.5	9.6
	Total Hospitalization days across entire country	11,820	565,670	577,490
Hospital Charges ($)	Mean	$749,370	$90,568	$95,047
	Standard error of mean	$69,766	$3,147	$3,343
	25^th^ percentile	$208,170	$13,886	$13,980
	Median– 50^th^ percentile	$528,388	$32,297	$32,770
	75^th^ percentile	$988,073	$78,910	$80,236
	Total hospitalization charges across entire country	$307,241,689	$5,425,018,316	$5,732,260,005

Multivariable logistic regression was used to examine the association between patient- and hospital-level variables and in-hospital mortality (Table 3), LOS (Table 4), and hospitalization charges (Table 5). After adjusting for confounders, ECMO use was associated with higher odds for in-hospital mortality (OR 11.15, 95% CI 6.57–18.92, *p* < 0.01), longer LOS (6.6 days, regression parameter estimate 0.5384, 95% CI 0.2401–0.8367, *p* < 0.001), and higher hospital charges ($510,523, regression parameter estimate 1.8518, 95% CI 1.6165–2.0871, *p* < 0.0001).

**Table 3:** Variables associated with in-hospital mortality.

Characteristics		Odds Ratio (95% CI)	*p*-value
ECMO	Required ECMO	11.15 (6.57–18.92)	<0.01*
Age	Each 1 year increase	0.97 (0.95–0.99)	0.01*
Sex	Male		Reference
	Female	0.92 (0.73–1.14)	0.44
Race	White		Reference
	Black	0.93 (0.69–1.26)	0.66
	Hispanic	0.75 (0.54–1.03)	0.08
	Asian/Pacific Islander	1.01 (0.57–1.81)	0.96
	Native American	0.71 (0.21–2.46)	0.59
	Other	1.06 (0.69–1.62)	0.80
Insurance	Private insurance		Reference
	Medicare	0.85 (0.22–3.29)	0.81
	Medicaid	1.23 (0.95–1.60)	0.12
	Uninsured	2.06 (1.17–3.64)	0.01*
	Other insurance	1.19 (0.75–1.89)	0.45
Comorbidities	No comorbidities		Reference
	Each additional comorbidity	1.74 (1.63–1.86)	<0.01*
Year of Hospitalization	2012	Reference	
	2013	0.88 (0.66–1.18)	0.39
	2014	0.90 (0.68–1.19)	0.45
Type of Admission	Emergent/urgent		Reference
	Elective	0.96 (0.60–1.53)	0.87
Type of Hospital	Urban non-teaching/rural		Reference
	Urban teaching	2.58 (1.66–4.00)	<0.01*
Region	West		Reference
	Northeast	1.46 (1.01–2.12)	0.05
	Midwest	1.02 (0.68–1.53)	0.91
	South	1.55 (1.11–2.15)	0.01*

* statistically significant for *p*-value < 0.05

**Table 4:** Variables associated with length of stay (LOS).

Characteristics		Regression Parameter Estimate (95% CI)	*p*-value
ECMO	Required ECMO	0.5384 (0.2401–0.8367)	<0.001*
Age	Each 1 year increase	-0.0221 (-0.0254 –-0.0189)	<0.0001*
Sex	Male		Reference
	Female	-0.0582 (-0.0903 –-0.0261)	<0.001*
Race	White		Reference
	Black	0.0743 (0.0167–0.1318)	0.0115*
	Hispanic	0.0679 (0.0160–0.1199)	0.0104*
	Asian/Pacific Islander	0.1107 (0.0145–0.2069)	0.0241*
	Native American	-0.0358 (-0.1776–0.1060)	0.6207
	Other	0.1126 (0.0331–0.1921)	<0.01*
Insurance status	Private insurance		Reference
	Medicare	-0.3389 (-0.5787 –-0.0991)	<0.01*
	Medicaid	0.0449 (0.0058–0.0841)	0.0244*
	Uninsured	0.0100 (-0.0864–0.1065)	0.8382
	Other insurance	0.0493 (-0.0505–0.1492)	0.3328
Comorbidities	No comorbidities		Reference
	Each additional comorbidity	0.2421 (0.2262–0.2581)	<0.0001*
Year of Hospitalization	2012		Reference
	2013	0.0115 (-0.0477–0.0708)	0.7028
	2014	-0.0660 (-0.1249 –-0.0070)	0.0285*
Type of Admission	Emergent/urgent		Reference
	Elective	0.0279 (-0.0426–0.0984)	0.4385
Type of Hospital	Urban non-teaching/rural		Reference
	Urban teaching	0.3459 (0.2949–0.3969)	<0.0001*
Region	West		Reference
	Northeast	-0.1091 (-0.1874 –-0.0308)	<0.01*
	Midwest	-0.1014 (-0.1631 –-0.0417)	<0.01*
	South	-0.0280 (-0.0919–0.0359)	0.3904

* statistically significant for *p*-value < 0.05

**Table 5:** Variables associated with hospital charges.

Characteristics		Regression Parameter Estimate (95% CI)	*p*-value
ECMO	Required ECMO	1.8518 (1.6165–2.0871)	<0.0001*
Age	Each 1 year increase	-0.0089 (-0.0132 –-0.0047)	<0.0001*
Sex	Male		Reference
	Female	-0.0975 (-0.1375 –-0.0574)	<0.0001*
Race	White		Reference
	Black	0.0977 (0.0198–0.1756)	<0.05*
	Hispanic	0.2138 (0.1385–0.2890)	<0.0001*
	Asian/Pacific Islander	0.2501 (0.1181–0.3820)	<0.001*
	Native American	-0.2115 (-0.4239–0.0009)	0.0510
	Other	0.1672 (0.0575–0.2769)	<0.01*
Insurance status	Private insurance		Reference
	Medicare	-0.4219 (-0.7253 –-0.1185)	<0.01*
	Medicaid	-0.0002 (-0.0514–0.0510)	0.9938
	Uninsured	-0.0425 (-0.1688–0.0838)	0.5095
	Other insurance	0.1193 (-0.0250–0.2637)	0.1051
Comorbidities	No comorbidities		Reference
	Each additional comorbidity	0.3962 (0.3754–0.4169)	<0.0001*
Year of Hospitalization	2012		Reference
	2013	0.0438 (-0.0589–0.1465)	0.4031
	2014	-0.0428 (-0.1448–0.0592)	0.4106
Type of Admission	Emergent/urgent		Reference
	Elective	-0.1313 (-0.2352 –-0.0274)	0.0133*
Type of Hospital	Urban non-teaching/rural		Reference
	Urban teaching	0.6730 (0.5943–0.7516)	<0.0001*
Region	West		Reference
	Northeast	-0.3925 (-0.5296 –-0.2554)	<0.0001*
	Midwest	-0.4992 (-0.6020 –-0.3964)	<0.0001*
	South	-0.4710 (-0.5770 –-0.3651)	<0.0001*

* statistically significant for *p*-value < 0.05

## Discussion

In our study, less than 1% of pediatric patients with sepsis received ECMO. We found that use of ECMO in children with sepsis is associated with considerable resource utilization but acceptable survival rate to discharge (59%). After adjusting for potential confounders, patients who received ECMO had an increased risk of in-hospital mortality, longer LOS, and higher hospitalization charges compared to those who did not.

ECMO utilization for sepsis has increased since its inclusion in the 2008 ACCM sepsis guidelines [16]. Although our study showed that ECMO was used in <1% of sepsis patients, a recent retrospective review of the Pediatric Health Information System database reported ECMO use in 4% of children with sepsis [16]. This discrepancy may reflect the fact that the higher rate was reported in a study of children’s hospitals, while our population included patients admitted at a diverse array of centers. It is possible that the smaller, non-children’s hospitals included in our study may be less likely to offer ECMO to pediatric patients than dedicated children’s hospitals, which may have more opportunities to utilize the therapy.

Our finding of 59% survival to discharge after ECMO is consistent with previous studies, which have shown survival rates of 80% in newborns and 30–50% in pediatric patients [5, 14–16, 22–25]. Extracorporeal Life Support Organization data report an overall ECMO survival rate of 61% for neonatal and pediatric patients, and retrospective pediatric studies have consistently reported survival rates of 46–56% [17, 22, 24, 26]. Taken in the context of these data, our study suggests that pediatric ECMO survival for patients with sepsis is comparable to that seen in patients receiving ECMO for other indications.

Unadjusted mean LOS in patients who received ECMO higher than in those who did not (28.8 versus 9.1 days). Recent studies of US pediatric sepsis admissions have reported a mean LOS of 9–17 days [8, 10, 27]. For pediatric ECMO patients, two retrospective analyses have reported mean LOS of 23–25 days [22, 26]. In our analysis, a number of factors were shown to be associated with LOS. ECMO use was associated with an average LOS 54% longer than that of the overall study population. This finding likely reflects both the tendency of these patients to have the greatest severity of illness and the fact that ECMO is usually accompanied by prolonged sedation and immobility, requiring extended de-escalation of support and extensive rehabilitation prior to discharge. Comparison of previous studies reporting LOS for sepsis and ECMO reveals a similar pattern [8, 10, 22, 26, 27]. Our study also showed that each additional year of age was associated with a 2% decrease in total LOS. This tendency toward longer LOS in younger children has been previously reported [9, 22]. Interestingly, we found that female patients had significantly shorter LOS. To our knowledge, this has not been reported previously, and future studies should examine the possible biological or physiologic basis for this discrepancy. We additionally found that each comorbidity was associated with a 24% increase in LOS, likely attributable to additional medical treatment and care coordination needs associated with chronic conditions. Our results are consistent with multiple previous studies showing an association between increasing comorbidities and longer LOS [6, 9, 27]. Finally, LOS in urban teaching hospitals was 35% longer than the mean for our population. Existing reports with similar findings have suggested that the highly specialized care and emphasis on rehabilitation and maximization of functional status prior to discharge may contribute to the longer LOS observed in these centers [27, 28].

The unadjusted mean hospital charges for patients placed on ECMO in our study were $749,370. Mean charges for patients not receiving ECMO were $90,568. Retrospective analyses of ECMO in pediatric patients have reported median hospitalization charges of $240,000-$690,000, with significant variation between hospitals [26, 29, 30]. The majority of available studies, however, report hospital costs, which have been shown to have a variable and inconsistent relationship to hospitalization charges and therefore make direct comparison with previous studies challenging [30]. In our analysis, hospitalization charges were also associated with a number of factors. In this study, charges for patients receiving ECMO were 185% higher than the average for our population. ECMO is a resource-intensive therapy associated with significant expense [22, 29, 30]. Although other studies have discussed ECMO costs and associated hospital charges [16, 29–31], our study provides an objective comparison of overall hospitalization charges for patients with sepsis who did and did not receive ECMO, offering insight into the financial implications of the therapy in these patients. Our analysis also showed an association between comorbid burden and hospital charges, with each additional diagnosis increasing charges by nearly 40%. This finding is consistent with previous studies [6, 27], and likely reflects the increased medical resources required to address the patients’ chronic as well as acute concerns. Hospitalization charges at urban teaching centers were 67% higher than those at urban non-teaching or rural hospitals. This discrepancy likely reflects a number of factors, including differences in illness severity, costs of subspecialty care, and greater technology utilization in teaching centers [27, 28]. Finally, we found a geographic difference in hospital charges, with higher charges in the western US than in other regions of the country. This regional variation is consistent with AHRQ data regarding inpatient charges, and has been demonstrated in previous pediatric ECMO research [30, 32].

Multivariable analyses revealed a number of factors associated with our outcomes of interest. Unsurprisingly, the strongest predictor of mortality was use of ECMO. ECMO is typically reserved for the sickest patients, for whom mortality is likely without the intervention. Although these patients have a significant risk of mortality even with the use of ECMO, our study shows that the majority who are placed on ECMO (59%) survive to discharge. Conversely, mortality rates decreased slightly with increasing patient age, consistent with existing reports indicating improved survival in older children [4, 9, 10]. Risk of mortality increased with each additional comorbidity, as had been shown previously [1, 4, 6, 14, 27]. Finally, odds of mortality were higher in children treated in urban teaching hospitals compared to non-teaching or rural hospitals. This finding has been documented previously and is likely secondary to the fact that smaller centers tend to transfer their sickest patients to tertiary teaching centers more equipped to deal with complicated patients, leading to a tendency for patients with more severe illnesses to be treated in teaching hospitals [27, 28, 33].

### Strengths of the study

To our knowledge, the present study is the largest and most representative cohort of hospitalized pediatric patients with sepsis for whom the use of extracorporeal membrane oxygenation and associated outcomes have been described. Available comparative studies are limited to single centers, smaller numbers, or older data. Our use of NIS data rather than single center experiences allows generalizability by ensuring that our study represents a diverse sampling of the US population. Currently, there is a relative lack of literature pertaining to the resource utilization in hospitalized children with sepsis needing ECMO. Our study begins to address this knowledge gap.

### Limitations of the study

The most notable limitations of our study are related to the nature of the dataset. First, the ability to identify and analyze patients with sepsis depends on the accuracy and thoroughness of information within the NIS administrative database. Use of administrative datasets to identify sepsis and outcomes has been widely reported [9, 10, 27, 28, 34]. Differences in coding practices or documentation that may exist among hospitals in this nationalized sample could potentially result in failure to identify or correctly categorize patients with sepsis, resulting in underestimation of prevalence. We used clinical classification software code for sepsis in the primary diagnosis field to identify those with sepsis; it is possible that sepsis was documented in the secondary diagnosis fields and hence could have been excluded in our analysis. Robust quality measures in collecting and reporting data attempt to minimize systematic variations in coding practices [21].

Second, NIS records provide information about hospital admissions, but do not include data regarding readmission rates, late mortality, or long-term health status. Post discharge data is not available in NIS dataset which precludes us from assessing the outcomes after discharge. The long-term outcomes of patients following ECMO are important considerations for the management of future patients, and these outcomes cannot be assessed based on the data contained within the NIS database.

Third, the study design limited our ability to assess all factors that might contribute to mortality and/or resource utilization in our population. Severity of sepsis is an important predictor of overall outcomes. The nature of the NIS dataset precludes us from assessing for the impact of the severity of sepsis. The surviving sepsis guidelines have been widely disseminated for clinical practice in Northern America and worldwide. It is known that clinical pathways for sepsis management vary amongst institute to institute and sometimes there is variation within an institute based on provider or patient specific variables. This level of granularity of data is not available in this otherwise large administrative NIS dataset. Nevertheless, ECMO in general is reserved for the most critically ill patients who fail standard of care therapy. It is hence reasonable to assume as such that those who needed ECMO for sepsis were the sickest of the cohort. Other factors such as the degree of organ dysfunction, for example, has also been shown to impact mortality in multiple previous studies of pediatric sepsis [6, 13, 16, 35]. Unfortunately, the degree of organ dysfunction or severity of illness at time of admission or at the time of ECMO deployment was not adjusted for in our analysis. Although, ECMO survival has been shown to be higher in centers with higher case volumes [26], we were unable to assess the impact of ECMO volume in our sample. Factors such as central cannulation vs peripheral cannulation, size of the cannula used, provider variables performing the cannulation and dynamic ECMO pump variables were not assessed in this study and should be the focus in future studies.

Fourth, although some previous studies have reported hospitalization charges, the majority describe hospital costs. The relation of hospitalization charges to hospital costs is variable. Comparing our financial data with previous studies is therefore challenging, and it is possible that use of hospital costs rather than hospitalization charges could provide a more clear representation of resource demands in our patient population.

Finally, due to the retrospective nature of the study, we are unable to assess causation. Although our results showed a number of variables that were associated with differences in mortality, LOS, and hospitalization charges, we are unable to assess whether or not modification of these variables would impact outcomes.

This study provides valuable data regarding current ECMO utilization and resource utilization in pediatric sepsis. Further research is needed to evaluate the impact of factors such as underlying illness severity and center volume on these outcomes. Additionally, information regarding the functional and neurological status of survivors would help clinicians make management decisions and counsel families as they evaluate treatment options. In the meantime, current data suggest that ECMO should be considered a viable strategy in children with refractory septic shock.

## Conclusion

Use of ECMO in children hospitalized for sepsis is associated with acceptable survival rates to discharge (59%) and should be considered a viable strategy in children with refractory septic shock. Further studies are needed to examine post discharge and neurocognitive outcomes in survivors.

## Abbreviations

ECMO: extracorporeal membrane oxygenation
LOS: length of stay
NIS: National (Nationwide) Inpatient Sample
ACCM: American College of Critical Care Medicine
AHRQ: Agency for Healthcare Research and Quality
HCUP: Healthcare Cost and Utilization Project

## References

[1] Carcillo JA. Pediatric septic shock and multiple organ failure. Crit Care Clin. 2003;19(3):413–40. Epub 2003/07/10. 10.1016/s0749-0704(03)00013-7 . 12848313

[2] Watson RS, Carcillo JA. Scope and epidemiology of pediatric sepsis. Pediatr Crit Care Med. 2005;6(3 Suppl):S3-5. Epub 2005/04/29. 10.1097/01.Pcc.0000161289.22464.C3 . 15857554

[3] Kawasaki T. Update on pediatric sepsis: a review. Journal of Intensive Care. 2017;5(1):47 10.1186/s40560-017-0240-1 28729906 PMC5518149

[4] Fleischmann-Struzek C, Goldfarb DM, Schlattmann P, Schlapbach LJ, Reinhart K, Kissoon N. The global burden of paediatric and neonatal sepsis: a systematic review. Lancet Respir Med. 2018;6(3):223–30. Epub 2018/03/07. 10.1016/S2213-2600(18)30063-8 . 29508706

[5] Weiss SL, Fitzgerald JC, Pappachan J, Wheeler D, Jaramillo-Bustamante JC, Salloo A, et al Global Epidemiology of Pediatric Severe Sepsis: The Sepsis Prevalence, Outcomes, and Therapies Study. Am J Respir Crit Care Med. 2015;191(10):1147–57. Epub 2015/03/04. 10.1164/rccm.201412-2323OC 25734408 PMC4451622

[6] Watson RS, Carcillo JA, Linde-Zwirble WT, Clermont G, Lidicker J, Angus DC. The Epidemiology of Severe Sepsis in Children in the United States. Am J Respir Crit Care Med. 2003;167(5):695–701. Epub 2002/11/16. 10.1164/rccm.200207-682OC . 12433670

[7] Davis AL, Carcillo JA, Aneja RK, Deymann AJ, Lin JC, Nguyen TC, et al American College of Critical Care Medicine clinical practice parameters for hemodynamic support of pediatric and neonatal septic shock. Crit Care Med. 2017;45(6):1061–93. Epub 2017/05/17. 10.1097/CCM.0000000000002425 . 28509730

[8] Ames SG, Davis BS, Angus DC, Carcillo JA, Kahn JM. Hospital Variation in Risk-Adjusted Pediatric Sepsis Mortality. Pediatr Crit Care Med. 2018 Epub 2018/02/21. 10.1097/pcc.0000000000001502 . 29461429 PMC5935525

[9] Hartman ME, Linde-Zwirble WT, Angus DC, Watson RS. Trends in the Epidemiology of Pediatric Severe Sepsis. Pediatr Crit Care Med. 2013;14(7):686–93. Epub 2013/07/31. 10.1097/PCC.0b013e3182917fad . 23897242

[10] Ruth A, McCracken CE, Fortenberry JD, Hall M, Simon HK, Hebbar KB. Pediatric Severe Sepsis: Current Trends and Outcomes From the Pediatric Health Information Systems Database. Pediatr Crit Care Med. 2014;15(9):828–38. Epub 2014/09/17. 10.1097/PCC.0000000000000254 . 25226500

[11] Schlapbach LJ, Straney L, Alexander J, MacLaren G, Festa M, Schibler A, et al Mortality related to invasive infections, sepsis, and septic shock in critically ill children in Australia and New Zealand, 2002–13: a multicentre retrospective cohort study. The Lancet Infectious Diseases. 2015;15(1):46–54. 10.1016/S1473-3099(14)71003-5 25471555

[12] Leclerc F, Leteurtre S, Duhamel A, Grandbastien B, Proulx F, Martinot A, et al Cumulative Influence of Organ Dysfunctions and Septic State on Mortality of Critically Ill Children. Am J Respir Crit Care Med. 2005;171(4):348–53. Epub 2004/11/02. 10.1164/rccm.200405-630OC . 15516535

[13] Weiss SL, Balamuth F, Hensley J, Fitzgerald JC, Bush J, Nadkarni VM, et al The Epidemiology of Hospital Death Following Pediatric Severe Sepsis: When, Why, and How Children With Sepsis Die. Pediatr Crit Care Med. 2017;18(9):823–30. Epub 2017/05/27. 10.1097/PCC.0000000000001222 28549024 PMC5581233

[14] Brierley J, Carcillo JA, Choong K, Cornell T, Decaen A, Deymann A, et al Clinical practice parameters for hemodynamic support of pediatric and neonatal septic shock: 2007 update from the American College of Critical Care Medicine. Crit Care Med. 2009;37(2):666–88. Epub 2009/03/28. 10.1097/CCM.0b013e31819323c6 19325359 PMC4447433

[15] Maclaren G, Butt W, Best D, Donath S, Taylor A. Extracorporeal membrane oxygenation for refractory septic shock in children: One institution's experience. Pediatr Crit Care Med. 2007;8(5):447–51. Epub 2007/08/19. 10.1097/01.PCC.0000282155.25974.8F . 17693912

[16] Ruth A, McCracken CE, Fortenberry JD, Hebbar KB. Extracorporeal therapies in pediatric severe sepsis: findings from the pediatric health-care information system. Crit Care. 2015;19:397 Epub 2015/11/11. 10.1186/s13054-015-1105-4 26552921 PMC4640405

[17] Barbaro RP, Paden ML, Guner YS, Raman L, Ryerson LM, Alexander P, et al Pediatric Extracorporeal Life Support Organization Registry International Report 2016. ASAIO J. 2017;63(4):456–63. Epub 2017/05/31. 10.1097/MAT.0000000000000603 28557863 PMC5626007

[18] MacLaren G, Butt W, Best D, Donath S. Central extracorporeal membrane oxygenation for refractory pediatric septic shock. Pediatr Crit Care Med. 2011;12(2):133–6. Epub 2010/05/11. 10.1097/PCC.0b013e3181e2a4a1 . 20453704

[19] Dellinger RP, Levy MM, Carlet JM, Bion J, Parker MM, Jaeschke R, et al Surviving Sepsis Campaign: International guidelines for management of severe sepsis and septic shock: 2008. Crit Care Med. 2008;36(1):296–327. Epub 2007/12/26. 10.1097/01.CCM.0000298158.12101.41 . 18158437

[20] Bokman CL, Tashiro J, Perez EA, Lasko DS, Sola JE. Determinants of survival and resource utilization for pediatric extracorporeal membrane oxygenation in the United States 1997–2009. J Pediatr Surg. 2015;50(5):809–14. 10.1016/j.jpedsurg.2015.02.042 25783363

[21] Healthcare Cost Utilization Project. HCUP-US NIS Overview. Rockville, MD: Agency for Healthcare Research and Quality; 2018 [cited 2018 Apr 21]. Available from: https://www.hcup-us.ahrq.gov/nisoverview.jsp.

[22] Bokman CL, Tashiro J, Perez EA, Lasko DS, Sola JE. Determinants of survival and resource utilization for pediatric extracorporeal membrane oxygenation in the United States 1997–2009. J Pediatr Surg. 2015;50(5):809–14. Epub 2015/03/19. 10.1016/j.jpedsurg.2015.02.042 . 25783363

[23] Chang TH, Wu ET, Lu CY, Huang SC, Yang TI, Wang CC, et al Pathogens and outcomes in pediatric septic shock patients supported by extracorporeal membrane oxygenation. J Microbiol Immunol Infect. 2017 Epub 2017/08/20. 10.1016/j.jmii.2017.07.012 . 28821378

[24] Mehta NM, Turner D, Walsh B, Zurakowski D, Betit P, Wilson J, et al Factors associated with survival in pediatric extracorporeal membrane oxygenation—a single-center experience. J Pediatr Surg. 2010;45(10):1995–2003. Epub 2010/10/06. 10.1016/j.jpedsurg.2010.05.028 . 20920718

[25] Meyer DM, Jessen ME. Results of Extracorporeal Membrane Oxygenation in Children With Sepsis. The Annals of Thoracic Surgery. 1997;63(3):756–61. 10.1016/s0003-4975(96)01272-6 9066397

[26] Jen HC, Shew SB. Hospital Readmissions and Survival After Nonneonatal Pediatric ECMO. Pediatrics. 2010;125(6):1217–23. Epub 2010/05/19. 10.1542/peds.2009-0696 . 20478938

[27] Odetola FO, Gebremariam A, Freed GL. Patient and Hospital Correlates of Clinical Outcomes and Resource Utilization in Severe Pediatric Sepsis. Pediatrics. 2007;119(3):487–94. Epub 2007/03/03. 10.1542/peds.2006-2353 . 17332201

[28] Hsu BS, Meyer BD, Lakhani SA. Financial, Resource Utilization and Mortality Impacts of Teaching Hospital Status on Pediatric Patients Admitted for Sepsis. Pediatr Infect Dis J. 2017;36(8):712–9. Epub 2016/12/30. 10.1097/INF.0000000000001526 . 28033241

[29] Barbaro RP, Boonstra PS, Moler FW, Davis MM, Prosser LA. Hospital-level variation in inpatient cost among children receiving extracorporeal membrane oxygenation. Perfusion. 2017;32(7):538–46. Epub 2017/05/04. 10.1177/0267659117702709 . 28466677

[30] Faraoni D, Nasr VG, DiNardo JA, Thiagarajan RR. Hospital Costs for Neonates and Children Supported with Extracorporeal Membrane Oxygenation. J Pediatr. 2016;169:69–75.e1. Epub 2015/11/09. 10.1016/j.jpeds.2015.10.002 . 26547402

[31] Harvey MJ, Gaies MG, Prosser LA. US and International In-Hospital Costs of Extracorporeal Membrane Oxygenation: a Systematic Review. Applied Health Economics and Health Policy. 2015;13(4):341–57. 10.1007/s40258-015-0170-9 25894740

[32] Karaca Z, Moore B. Geographic Variation in Hospital Inpatient List Prices in the United States, 2013 HCUP Statistical Brief #209. Rockville, MD: Agency for Healthcare Research and Quality; 8 2016. 27748097

[33] Hsu BS, Schimelpfenig M, Lakhani S. Comparison of Transferred Versus Nontransferred Pediatric Patients Admitted for Sepsis. Air Med J. 2016;35(1):43–5. Epub 2016/02/10. 10.1016/j.amj.2015.09.005 . 26856659

[34] Jolley RJ, Sawka KJ, Yergens DW, Quan H, Jette N, Doig CJ. Validity of administrative data in recording sepsis: a systematic review. Crit Care. 2015;19:139 Epub 2015/04/19. 10.1186/s13054-015-0847-3 25887596 PMC4403835

[35] Kutko MC, Calarco MP, Flaherty MB, Helmrich RF, Ushay HM, Pon S, et al Mortality rates in pediatric septic shock with and without multiple organ system failure. Pediatr Crit Care Med. 2003;4(3):333–7. Epub 2003/07/02. 10.1097/01.PCC.0000074266.10576.9B . 12831416

